# Species identification of phlebotomine sandflies using deep learning and wing interferential pattern (WIP)

**DOI:** 10.1038/s41598-023-48685-2

**Published:** 2023-12-04

**Authors:** Arnaud Cannet, Camille Simon-Chane, Aymeric Histace, Mohammad Akhoundi, Olivier Romain, Marc Souchaud, Pierre Jacob, Darian Sereno, Petr Volf, Vit Dvorak, Denis Sereno

**Affiliations:** 1 Direction des Affaires Sanitaires et Sociales de la Nouvelle-Calédonie, Nouméa, France; 2 ETIS UMR 8051, Cergy Paris University, ENSEA, CNRS, 95000 Cergy, France; 3 Cergy Paris University, Cergy, France; 4 Parasitology-Mycology, Hopital Avicenne, AP-HP, Bobigny, France; 5 Univ. Bordeaux, CNRS, Bordeaux INP, LaBRI, UMR 5800, 33400 Talence, France; 6 InterTryp, Univ Montpellier, IRD, CIRAD, Infectiology, Entomology and One Health Research Group, Montpellier, France; 7 Laboratory of Vector Biology, Department of Parasitology, Faculty of Science, Charles University, Prague, Czech Republic; 8 MIVEGEC, Univ Montpellier, CNRS, IRD, Montpellier, France

**Keywords:** Zoology, Entomology

## Abstract

Sandflies (Diptera; Psychodidae) are medical and veterinary vectors that transmit diverse parasitic, viral, and bacterial pathogens. Their identification has always been challenging, particularly at the specific and sub-specific levels, because it relies on examining minute and mostly internal structures. Here, to circumvent such limitations, we have evaluated the accuracy and reliability of Wing Interferential Patterns (WIPs) generated on the surface of sandfly wings in conjunction with deep learning (DL) procedures to assign specimens at various taxonomic levels. Our dataset proves that the method can accurately identify sandflies over other dipteran insects at the family, genus, subgenus, and species level with an accuracy higher than 77.0%, regardless of the taxonomic level challenged. This approach does not require inspection of internal organs to address identification, does not rely on identification keys, and can be implemented under field or near-field conditions, showing promise for sandfly pro-active and passive entomological surveys in an era of scarcity in medical entomologists.

## Introduction

Sandfly insects belong to the order Diptera, family Psychodidae. They are medical and veterinary important vectors of diverse viral, bacterial, and protozoan pathogens. Leishmaniases, caused by protozoan parasites of the genus *Leishmania* (Trypanosomatida: Trypanosomatidae), occur in large areas, mainly the tropics and subtropics, recently emerging into new regions due to climatic and environmental changes^1–4^. Leishmanioses are among most important Neglected Tropical diseases with their endemic distribution including more than 98 countries and territories where over 350 million people are at risk for infection and 12 million individuals affected annually. Moreover, canine leishmaniasis is a severe veterinary problem, with an estimated 2.5 million dogs infected only in the Mediterranean basin^5^. Besides being principal vectors of most *Leishmania* species, phlebotomine sandflies are also involved in the transmission of viruses belonging to *Rhabdoviridae*, *Flaviviridae*, *Reoviridae*, *Peribunyaviridae,* and *Phenuiviridae* families. Among these, *Phenuiviridae* (Bunyavirales), encompassing the Phlebovirus genus, is often identified in sandflies and threatens human health^6,7^. Among pathogenic bacteria trasmitted by sandflies, *Bartonella bacilliformis*, a causative agent of Carrión's disease in rural Andean areas of Peru and Ecuador, shall be mentioned^8^.

Over 900 sandfly species are recognized and formally described from Old and New World^1^. As only some species have a vectorial capacity to contribute to parasite transmission (118 suspected and 47 proven vectors), from a perspective of medical entomology, it is of uppermost importance to accurately identify phlebotomine sandflies. Only species belonging to *Phlebotomus* in the Old World and in the New World, various genera, including *Lutzomyia Migonemyia…* in the New World, are regarded as proven vectors of human and veterinary pathogens. However, field and laboratory evidence supports some species of the *Sergentomyia* genus as potential vectors' role in *Leishmania* and viruses’ transmission^6,9,10^.

Phlebotomine sandfly morphological identification has always been challenging, particularly at the specific and sub-specific levels, because of variations in criteria and methods, morphological similarities between species, the inadequacy of descriptions, and the massive increase in the number of sandfly species described. Besides these limitations, the need to examine internal structures (arrangements of aedeagi and appendages in males, morphology of spermathecae in females) or external structures prone to damage during trapping and sample handling (e.g., the structure of the male genitalia, wing venation, and antennal and the palpal formula) is time-consuming and puzzled species identification. In addition, it is devoted to a limited and currently decreasing number of skilled specialists. Therefore, implementing new, affordable, and easy-to-handle methods for accurate sandfly identification is crucial from an entomological and medical survey perspective.

Wing Interference Patterns (WIPs) have received attention for their taxonomic potential^11–13^. The thin-film interference occurring on the wings' transparent membrane allows the formation of a colored pattern. These WIPs significantly vary among specimens belonging to different species but moderately between specimens for the same species or between sexes. Unlike the angle-dependent iridescence effect of a flat film, the newton color series displayed is proportional to the thickness of the wing membrane at any given point, wing structures acting as diopters ensuring the WIPs appear essentially non-iridescent^12^. Deep learning (DL), a branch of machine learning (ML) and artificial intelligence (AI), has achieved outstanding results on several complex cognitive tasks, matching or beating those provided by human performance. It has proven helpful for tasks such as image and speech recognition, natural language processing, and object detection^14^. Recently, we have probed the capability of DL in classifying WIP pictures taken from wings of (Glossinidae Theobald, 1903)^15^ and (Culicidae Meigen 1818)^16^, two medically important dipteran families. Here, we investigate the reliability and specificity of such a method for Old World Phlebotominae species diagnostic and provide some clues that WIPs can be detected on New World species and, therefore, would be amenable.

## Material and methods

### Specimen selection and storage

The database of WIP from Psychodidae insects, comprising 1673 pictures, gathers samples belonging to the Phlebotominae family in the majority from well-established laboratory breeds, limiting potential intrapopulation WIP variations, but also from specimens collected *in natura* whose identification was performed at the time of their catch with available regional identification keys. Laboratory-reared representatives were provided using a standard method of sand fly breeding^17^. The description of the samples used in this study is given in Table 1.

**Table 1:** Psychodidae Phlebotominae samples in the database.

	Med	Or	Year	N	Country^&^	Bred origin	Provider
*Phlebotomus (Phlebotomus) argentipes*	Yes	C	2014	**192**	203	356	P Volf and V Dvorak
*Phlebotomus (Phlebotomus) duboscqi*	Yes	C	2018	**150**	203	686	P Volf and V Dvorak
*Phlebotomus (Phlebotomus) papatasi*	Yes	C	2018	**135**	203	196	P Volf and V Dvorak
*Phlebotomus (Laroussius) ariasi*	Yes	W	2012	**20**	250- Nice		A Cannet
*Phlebotomus (Larroussius) orientalis*	Yes	C	2014	**116**	203	231	P Volf and V Dvorak
*Phlebotomus (Larroussius) perfilliewi*	Yes	W	2018	**4**	364, NK		M Akhoundi
*Phlebotomus (Larroussius) perniciosus*	Yes	C	2018	**180**	203	380	P Volf and V Dvorak
*Phlebotomus (Larroussius) tobbi*	Yes	C	2014	**163**	203	792, Adana	P Volf and V Dvorak
*Phlebotomus (Transsphlebotomus) mascittii*	No	W	2012	**4**	250- Nice		A Cannet
*Phlebotomus (Paraphlebotomus) sergenti*	Yes	C	2014	**226**	203	792, Urfa	P Volf and V Dvorak
*Phlebotomus (Adlerius) arabicus*	Yes	C	2018	**82**	203	376	P Volf and V Dvorak
*Lutzomyia (Lutzomyia) longipalpis*	Yes	C-W	2014	**167–5**	203, 250-French guiana	76, Jacobina	P Volf and V Dvorak, A Cannet
*Lutzomyia (Lutzomyia) migonei*	Yes	C	2018	**117**	203	76, Baturité	P Volf and V Dvorak
*Migonemyia (Migonemyia) migonei*							
*Lutzomyia (Trychophoromyia) ubiquatilis*	No	W	2014	**1**	250-French guiana		A Cannet
*Trchophoromyia ubiquatilis*							
*Lutzomyia (Trychophoromyia) ininii*	Yes	W	2014	**4**	250-French guiana		A Cannet
*Trychophoromyia ininii*							
*Sergentomyia (Sergentomyia) schwetzi*	No	C	2018	**100**	203	231	P Volf and V Dvorak
*Sergentomyia (Sergentomyia) minuta*	No	W	2012	**7**	250-Nice		A Cannet

^&^ISO 3166–1 country code available at (https://www.atlas-monde.net/codes-iso/).

*Med* medical importance, *0r* the sample's origin, *W* wild, *C* colony, *N* number of samples processed, *NK* not known.

Significant values are in bold.

### Image acquisition and database construction

**Figure 1:**
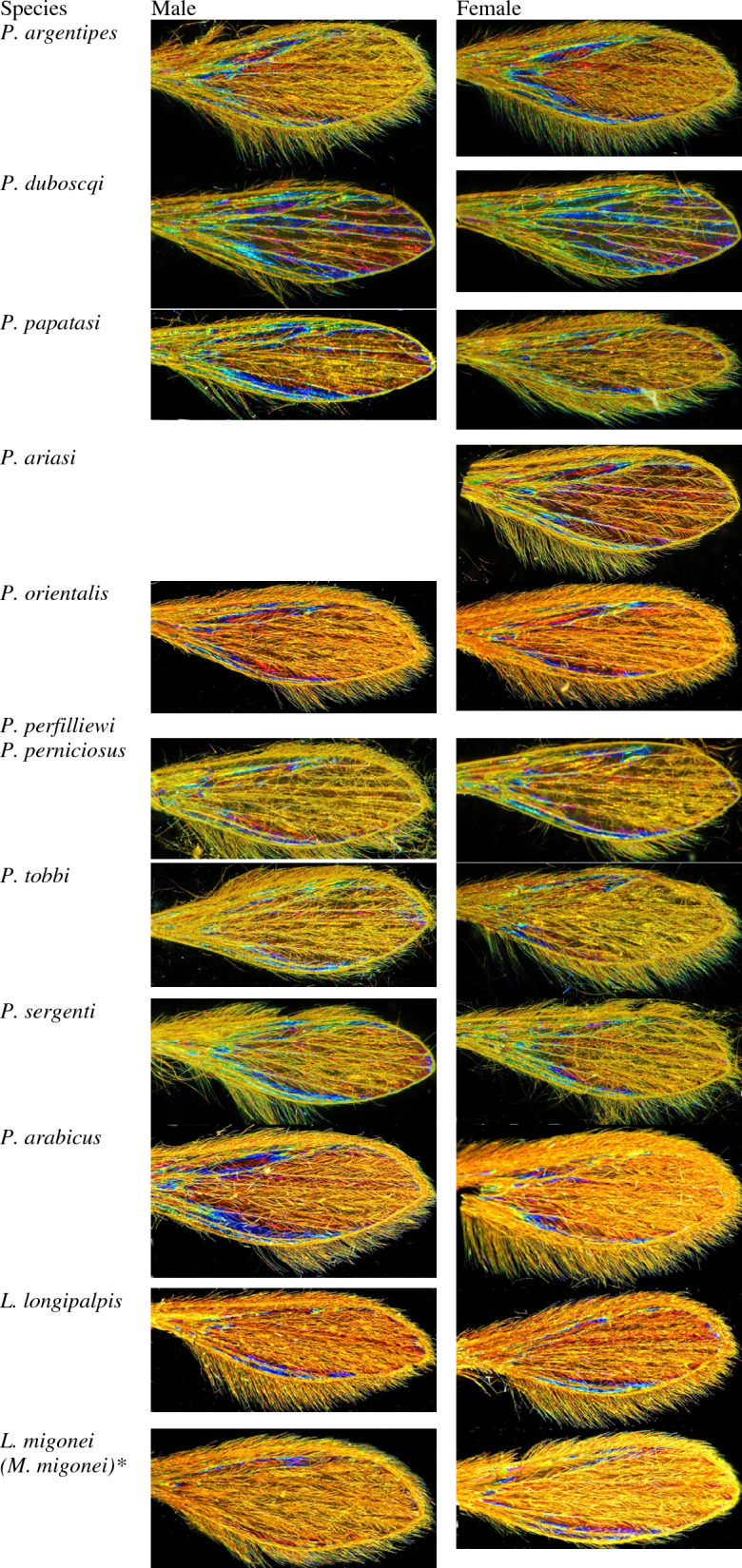
Examples of pictures included in the training dataset. *According to the updated taxonomy of New World sandflies.

The protocol used to capture sandfly WIPs was as already described for *Glossina sp.* WIPs acquisition^15^. Briefly, dissected wings were deposited on a glass slide and covered by a small cover slide. A Keyence™ VHX 1000 microscope, using the VH-Z20r camera, and a Keyence VH K20 adapter set for an illumination incidence of 10° were used. All pictures were enlarged to a maximal occupancy, and the High Dynamic Range (HDR) function was used for all photos. Shots were then filled in a database with their taxonomic information, sex, date of capture, country of collection, and name of the entomologist that has undergone morphological identification. All pictures were filled in the database. See Fig. 1 for an example of images gathered during the study.

### Collected dataset, image pre-processing, and dataset splitting for training/learning and validation.

**Figure 2:**
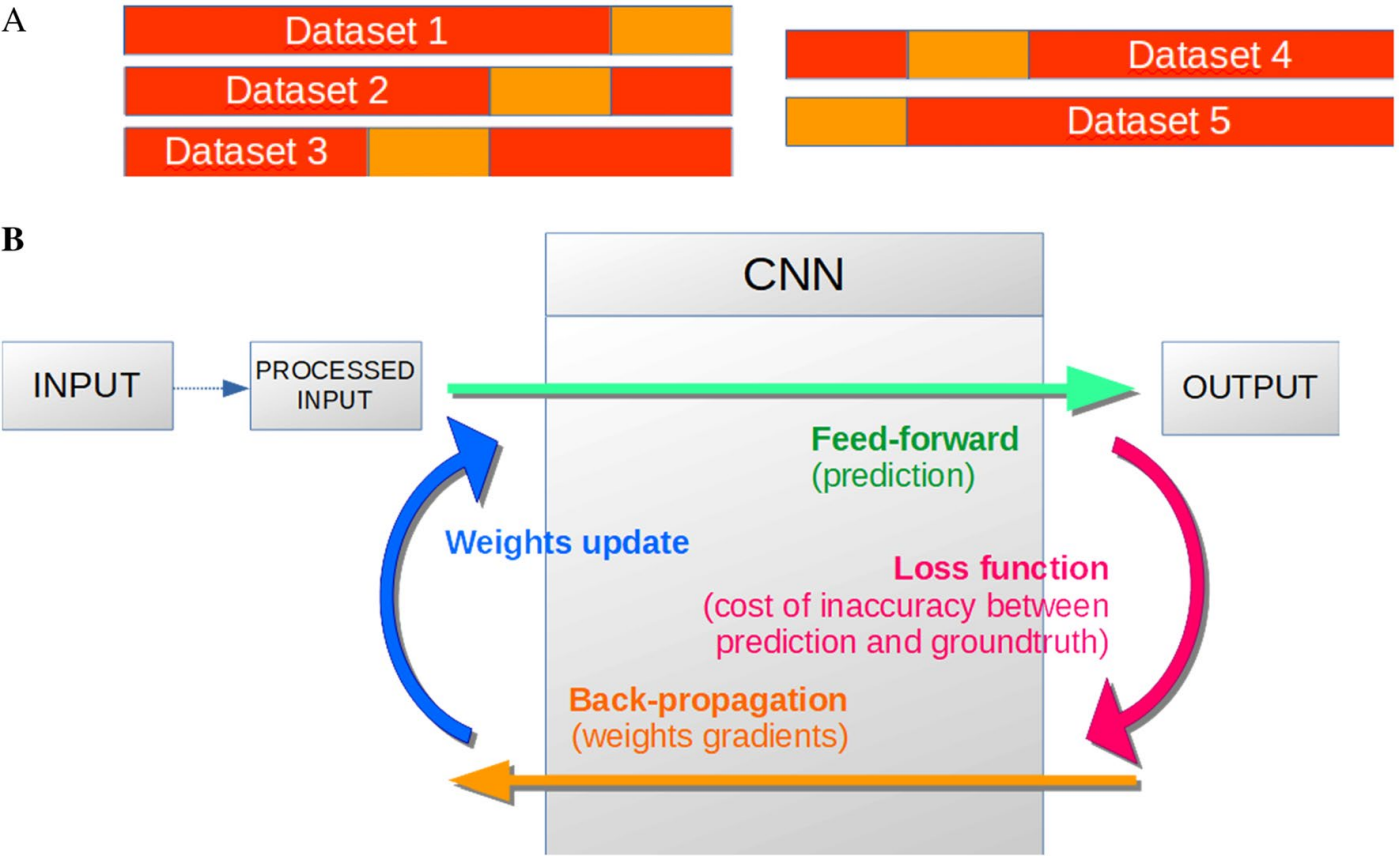
(**A**) Schematic representation of the dataset splitting for learning (red) and testing (orange), (**B**) representation of the pipeline process developed using the Convolutional Neural Network (CNN) approach. Two steps predict the class of a given Phlebotominae WIP images: extracting hierarchical features (Convolutional layer) and classifying these features (Fully-connected layer and softmax layer). These feature maps are used for visualization by weighting them with channel-wise averaged gradients from Cannet et al.^15^.

The Phlebotomine dataset includes 1673 pictures of 17 sandfly species^18^. Underrepresented sandfly species (less than ten samples/pictures) were discarded from the training dataset to prevent overfitting. Processed images were then resized to 256 width and 116 height pixels, and pixel values were normalized within the (0,1) range. The dataset was prepared for k-fold cross-validation, with k = 5, shuffled randomly, and partitioned into k equal-size subsets having a similar class distribution. A separately learned classifier was evaluated for each subgroup using kth of the whole dataset for validation and the remaining k-1 as training data (see Fig. 2A for illustration).

This strategy allowed measuring the mean accuracy of the five distinct classifiers. Among all existing machine learning methods, Deep Convolutional Neural Networks and their different architectures have shown in the last decade to be the most adapted for image classification. A pipeline overview of the complete training procedure using CNN is shown in Fig. 2B.

### Training of the neural network (CNN)

CNN architecture of MobileNet^19^, ResNet^20^, and YOLOv2^21^ were deemed for automatically classifying sandfly specimens with the dataset. Compared to classic Deep Learning, ours is more compact to cope with the specificity of our dataset in terms of size; therefore, thinner image recognition and classification architecture were developed to consider its reduced size. Inspired by MobileNet, the first takes advantage of depth-wise convolution^19^, with only one depth-wise convolution per layer of the CNN architecture that reduces the complexity and number of features extracted. A batch normalization was set to speed up and stabilize the training process^22^. In addition to this first compact CNN architecture, two interconnected layers like VGG^23^ for YOLOv2 were applied with a DarkNet-19^21^ architecture with 1 or 2 scales less than the original network. For clarity, we called them DarkNet-9 (8 convolution layers and one classification layer) and DarkNet-14 (13 convolution layers and one classification layer). We also reproduced the ResNet18 architecture from He and collaborator^20^ and trained it from random initialization. Even if this architecture seems too “deep” (may lead to overfitting) compared to our other architectures, the intrinsic properties of ResNet18, residual connections, allow convergence of the training procedure. We used a standard approach (shallow approach) based on extracting SURF descriptors (an efficient implementation of the classic SIFT descriptors), a Bag of Features (BoF) representation using a 4000 codewords dictionary, and an SVM with a standard polynomial kernel similar to it was proposed in Sereno et al.^24^.

## Results and discussion

To thoroughly investigate the proposed method's accuracy, we test its capability to correctly assign sandfly specimens at various taxonomic levels: subfamily, genus and subgenus, and species.

### Test for accuracy at the family/subfamily taxonomic level

The accuracy of the classifier was tested at various taxonomic levels ranging from the family (*Psychodidae*) to the genera (*Phlebotomus*, *Lutzomyia (Lutzomyia & Migonemyia,* if taking into account revised taxonomy^25,26^*)*, *Sergentomyia*) and the species level (12). The *Psychodidae* family encompasses about 2600 species; however, only specimens belonging to the Phlebotominae subfamily are included in the dataset due to their medical importance as pathogen vectors. We first explored the training classifier accuracy on the *Phlebotomine* dataset and other non-Psychodidae specimens from Calliphoridae, Culicidae, Glossinidae, Muscidae, and Tabanidae datasets^18^. We trained the CNN on such a combination to improve the model's accuracy. The dataset was filled with 1673 pictures of Phlebotomine WIPs. Still, five species were unsatisfactorily covered in terms of WIP pictures and were discarded from the training dataset of the Phlebotomine subset. Using this pictures-set, we ascertain the accuracy of the process to discriminate the Psychodidae family from other non-Psychodidae. From our dataset and method, the automatic classification process accuracy is an astonishing 99.8% (Table 2). Knowing that the wing size doesn’t belong to the descriptor selected during the training process, our classification accuracy would rely on other descriptors more specific to the WIPs.

**Table 2:** Psychodidae vs. non-Psychodidae classification accuracy.

		Predicted	
		*Psychodidae*	Non-*Psychodidae dipteran*
Truth	*Psychodidae ****(331)***	99.7%	1
	Non-*Psychodidae dipteran ****(685)***	1	99.8%

Number of pictures in bold.

### Test for accuracy at the genus taxonomic level

Sandfly taxonomy has complex and still ongoing evolution. A conservative and simplified approach recognizes six main genera: three in the Old World (*Phlebotomus*, *Sergentomyia,* and *Chinius*) and three in the New World (*Lutzomyia*, *Brumptomyia,* and *Warileya*)^1^. Although a revision of the New World genera was recently proposed^1,26^, in this study, since we refer to this conservative taxonomy^27^, we still added the information dealing with the revision of the New World sandfly taxonomy to highlight changes. Therefore*, L. migonei* specimens are gathered with those of *L. longipalpis* in the analysis and the accuracy computation. We have also focused on the genera that harbor proven or suspected vectors and thus are most relevant for human or veterinary medicine. Our dataset contains pictures documenting three genera if we refer to Akhoundi and Coll^1^ (*Phlebotomus*, *Lutzomyia,* and *Sergentomyia*), and four if we refer to the revised taxonomy^25,26^, clearly more samples are required to address this question on New World sandfly fauna. At the genus level, our classification accuracy was always > 90% (Table 3).

**Table 3:** Classification accuracy of genera.

	Genus	Predicted			
		*Phlebotomus*	*Lutzomyia (Lutzomyia and Migonemyia)*	*Sergentomyia*	Non-Psychodidae dipteran
Truth	*Phlebotomus* **(254)**	*98.0*	1.6	0.0	0.4
	*Lutzomyia (Lutzomyia and Migonemyia)*** (58)**	5.2	*93.1*	1.7	0.0
	*Sergentomyia* **(20)**	0.0	10.0	*90.0*	0.0
	Non-*Psychodidae dipteran* **(686)**	0.1	0.0	0.0	*99.9*

Number of pictures in bold.

Significant values are in italics.

### Test for accuracy at the subgenus taxonomic level

To further investigate the taxonomic congruence of our methodology with the already proposed one, we assess its classification reliability at the subgenus level. At the generic level, the subgenera of sandflies have been intensively studied over many decades, with taxonomists providing varying views about their number and designation. The genus *Phlebotomus* currently encompasses 13 subgenera*.* For the genus *Sergentomyia*, ten subgenera are proposed. The *Chinus* genus is not further divided into subgenera. The taxonomic subdivisions in Neotropical Phlebotominae are rather complex and remain debatable^26,28^. A checklist of American sandflies is available^25^. We use the classification provided by Akhoundi et al.^1^. Our dataset does not fully cover the biodiversity of sandflies, particularly for New World sandfly species, at the subgeneric level; however, we provide data on four subgenera of the genus *Phlebotomus*, namely *Adleriu*s, *Larroussius*, *Paraphlebotomus* and *Phlebotomus*, that in total harbor 30 species proven or suspected as vectors or many human-infecting *Leishmania*^4^. At the subgenus level, the classification accuracy computed remains high, consistently above 80% (Table 4). Higher confusion occurs between the *Adlerius* and *Laroussius* subgenera, which are regarded as phylogenetically close, than the *Sergentomyia* and New World (*Lutzomyia* and *Migonemyia)* ones.

**Table 4:** Classification accuracy of subgenera.

	Subgenus	Predicted						
		*Adlerius*	*Laroussius*	*Paraphlebotomus*	*Phlebotomus*	*Lutzomyia (Lutzomyia and Migonemyia)*	*Sergentomyia*	Non-*Psychodidae dipteran*
Truth	*Adlerius* **(17)**	*82.3*	11.8	0.0	5.9	0.0	0.0	0.0
	*Laroussius* **(96)**	1.0	*88.6*	0.0	6.3	3.1	0.0	1.0
	*Paraphlebotomus* **(45)**	0.0	4.4	*88*.*9*	6.7	0.0	0.0	0.0
	*Phlebotomus* **(96)**	0.0	0.0	0.0	*99*.*0*	1.0	0.0	0.0
	*Lutzomyia (Lutzomyia and Migonemyia)* **(58)**	0.0	3.5	0.0	1.7	*93*.*1*	1.7	0.0
	*Sergentomyia* **(20)**	0.0	0.0	0.0	0.0	10.0	*90*.*0*	0.0
	non-*Psychodidae dipteran* **(686)**	0.0	0.1	0.0	0.0	0.0	0.0	*99*.*9*

Number of pictures in bold.

Significant values are in italics.

### Test for automatic classification of the 12 sandfly species filled in the dataset

At the species level, our data set is filled with pictures of 17 species, but only 12 provided enough images to encompass a training process. Even if limited in terms of species richness, considering the vast number of sandfly species described (900–1000), our dataset is composed of primary proven vectors of pathogens causing cutaneous and visceral leishmaniasis in the New and Old World, which highlighted the interest for medical entomology purposes. Among the 12 species in the dataset that have undergone a learning process, the best accuracy score was for *P. papatasi* (100%), the proven vector of *L. major* (an agent of cutaneous leishmaniasis). The lower recorded accuracy score (77.8%) is computed for *P. perniciosus*, a proven vector of *L. infantum* (an agent of visceral leishmaniasis). The overall accuracy scores remain astonishing and are always higher than 77% for all the species filled in the database (Table 5). The exactness of the method to assign sandfly species must now be probed on a larger *in natura* collected sandfly sample covering a wider geographic area, including New World species. In addition, with the methods proposed, WIPs variation at the populational level can be investigated with a proper sampling strategy.

**Table 5:**
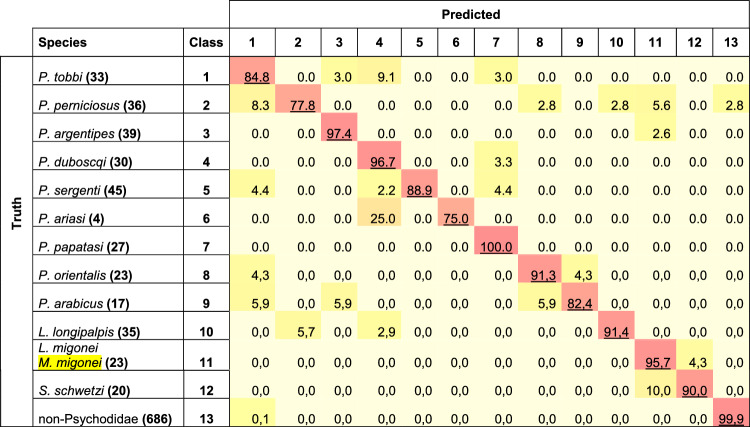
Accuracy score for specimens of the 12 Phlebotominae filled in the database.

Number of pictures in bold.

Morphological species identification remains a golden standard in sandfly taxonomy; however, it is prone to various limitations, including compromised state of decisive structures in the field-collected specimens, intraspecific variability among populations, laborious sample preparation and declining entomological expertise among taxonomists. Hence, alternative molecular approaches are gradually applied, namely DNA sequencing (DNA barcoding)^29^ or MALDI-TOF protein profiling^30–35^. These methods, however, also have their limitations. It was demonstrated that reference DNA sequences for sandflies currently cover less than 50% of the subfamily species diversity, and, depending on the sandfly group or genus, different markers rather than a universal set are applied^26,36^. Moreover, despite increasing affordability and decreasing costs per analysis, sequencing is still not always available in some endemic countries and requires considerable expertise. MALDI-TOF protein profiling, a mass spectrometry method, provides a time- and cost-effective alternative as the sample preparation is quick and cheap. However, the required machinery may be prohibitively expensive and not always readily available to medical entomologists. So far, only in-house databases of sandfly reference protein spectra have been established, further limiting the applications of this approach. In addition, the interoperability of MALDI-TOF requires a standardized procedure in the conservation of samples, the choice of the adult specimen body part or even the trapping method^37^, and the standardization of procedures for preparation and reproducibility between instruments and homemade databases is desirable^33^. Hence, an alternative method for species identification of adult sandflies performed under conditions that do not allow costly and highly sophisticated infrastructures is highly desirable.

The application of Deep learning leads to robust results in terms of classification performance. The proposed method has the potential to be used in real-life scenarios since the proposed architecture ends up with a good compromise compared to other methodologies reviewed in Cannet et al.^15^. Future development and technical implementation of this methodology include strengthening the database in terms of Phlebotomine species and population representation, the use of GANs (Generative adversarial network) allowing to fill up the database with new species, even with a low number of representatives. From an application point of view, previous works^15,16,24^ and this study add evidence to the generic potential of the method for dipteran insect identification. Implementing a SaaS platform would offer a complete service for remotely localized computers with an internet connection.

## Conclusions

During field entomological surveys, most routine sandfly identification involves diagnostic criteria, requiring dissections and slide-mounting to examine internal organs. An alternative morphological approach that surpasses the need for molecular analyses will be to develop computer vision relying on visual characters of taxonomic interest to assign taxon names. Therefore, using WIPs as a valuable taxonomic marker in conjunction with DL will help address challenges concerning sandfly identification. Further analyses of field-caught specimens of a more significant part of sandfly biodiversity will be needed to increase the method’s accuracy.

## Data Availability

The source code is publicly available on GitHub, with a direct URL: https://github.com/marcensea/diptera-wips.git.

## Code availability

Dataset is available with a direct URL: 10.6084/m9.figshare.22083050.v4.
